# Estrogen Receptor Function: Impact on the Human Endometrium

**DOI:** 10.3389/fendo.2022.827724

**Published:** 2022-02-28

**Authors:** Kun Yu, Zheng-Yuan Huang, Xue-Ling Xu, Jun Li, Xiang-Wei Fu, Shou-Long Deng

**Affiliations:** ^1^National Engineering Laboratory for Animal Breeding, Key Laboratory of Animal Genetics, Breeding and Reproduction, Beijing Key Laboratory for Animal Genetic Improvement, College of Animal Science and Technology, China Agricultural University, Beijing, China; ^2^Chelsea and Westminster Hospital, Department of Metabolism, Digestion and Reproduction, Imperial College London, London, United Kingdom; ^3^Department of Reproductive Medicine, The First Hospital of Hebei Medical University, Shijiazhuang, China; ^4^National Health Commission of China (NHC) Key Laboratory of Human Disease Comparative Medicine, Institute of Laboratory Animal Sciences, Chinese Academy of Medical Sciences and Comparative Medicine Center, Peking Union Medical College, Beijing, China

**Keywords:** endometrium, estrogen receptor α, estrogen receptor β, G-protein-coupled estrogen receptor, human

## Abstract

The physiological role of estrogen in the female endometrium is well established. On the basis of responses to steroid hormones (progesterone, androgen, and estrogen), the endometrium is considered to have proliferative and secretory phases. Estrogen can act in the endometrium by interacting with estrogen receptors (ERs) to induce mucosal proliferation during the proliferative phase and progesterone receptor (PR) synthesis, which prepare the endometrium for the secretory phase. Mouse knockout studies have shown that ER expression, including ERα, ERβ, and G-protein-coupled estrogen receptor (GPER) in the endometrium is critical for normal menstrual cycles and subsequent pregnancy. Incorrect expression of ERs can produce many diseases that can cause endometriosis, endometrial hyperplasia (EH), and endometrial cancer (EC), which affect numerous women of reproductive age. ERα promotes uterine cell proliferation and is strongly associated with an increased risk of EC, while ERβ has the opposite effects on ERα function. GPER is highly expressed in abnormal EH, but its expression in EC patients is paradoxical. Effective treatments for endometrium-related diseases depend on understanding the physiological function of ERs; however, much less is known about the signaling pathways through which ERs functions in the normal endometrium or in endometrial diseases. Given the important roles of ERs in the endometrium, we reviewed the published literature to elaborate the regulatory role of estrogen and its nuclear and membrane-associated receptors in maintaining the function of endometrium and to provide references for protecting female reproduction. Additionally, the role of drugs such as tamoxifen, raloxifene, fulvestrant and G-15 in the endometrium are also described. Future studies should focus on evaluating new therapeutic strategies that precisely target specific ERs and their related growth factor signaling pathways.

**Graphical Abstract:**
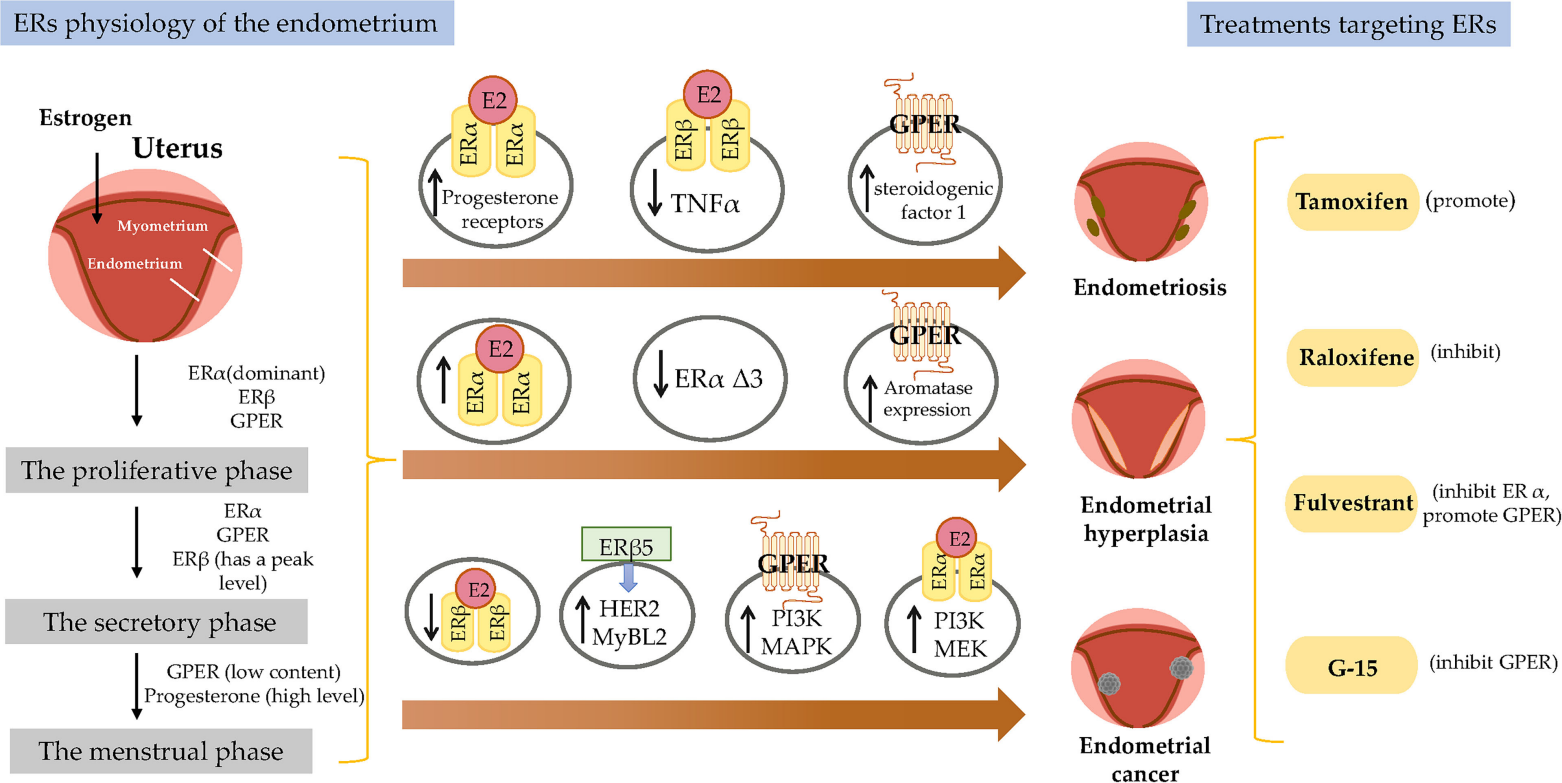


## Introduction

The endometrium is the primary target tissue for estrogen. The main function of the endometrium is to prepare for implantation and to maintain the pregnancy after embryo implantation. Estrogen exerts a critical influence on female reproduction *via* the two main classical estrogen receptors (ERs), ERα and ERβ, and perhaps through G-protein-coupled estrogen receptor (GPER; formerly GPR30) (1). Recent descriptions of the phenotypes of ER-knockout mice have also revealed key roles of estrogen signaling in the endometrium. Estrogen acts through ERs that are associated with the onset and maintenance of disease and tumor events, particularly in uterine tissue. This review focuses on recent advances in our knowledge of the role of ERs in the endometrium, animal models of ER deficiency in the uterus, and how ERs are involved in endometrial diseases and pharmacological treatments.

## ER Activation

**Figure 1:**
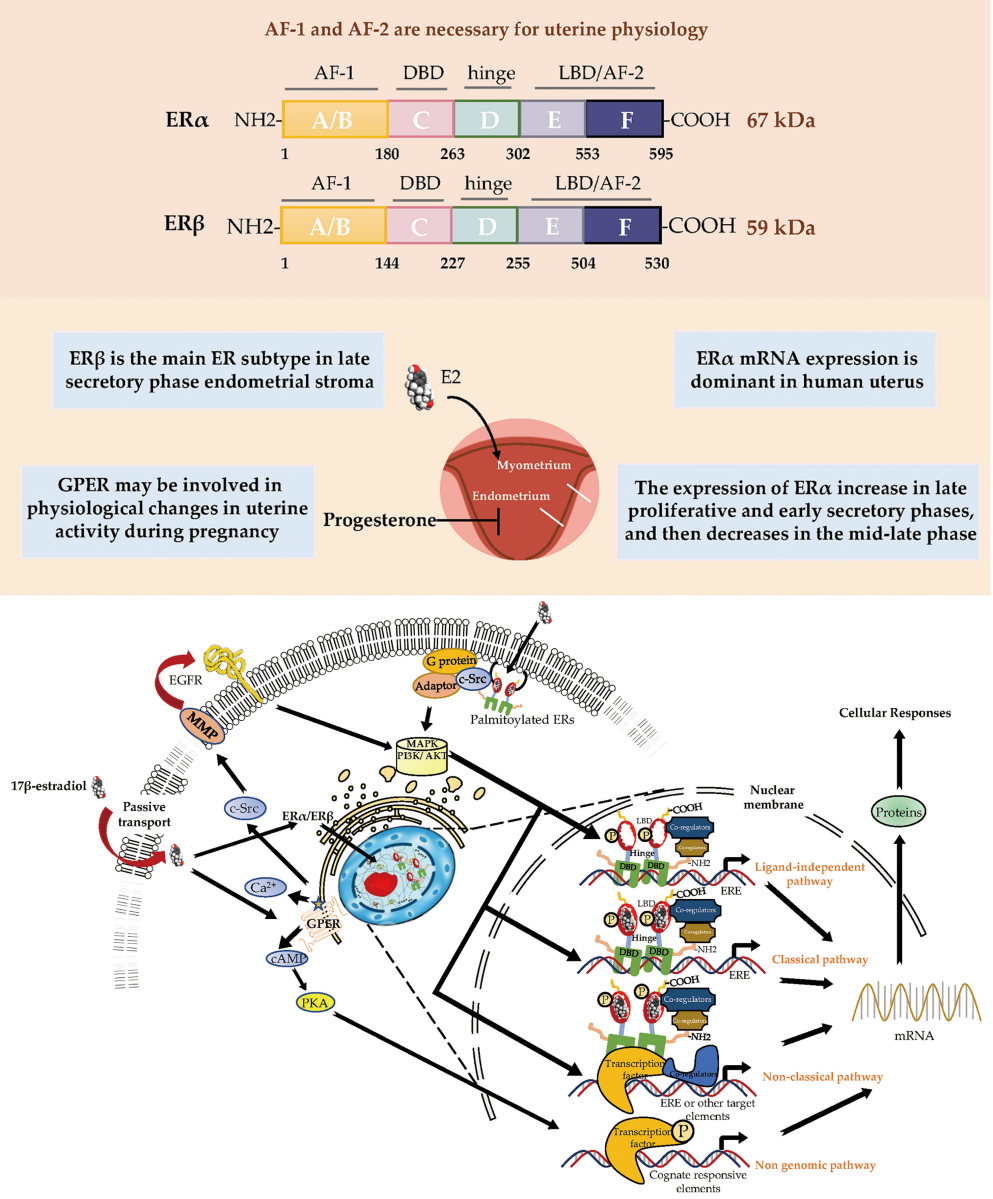
Estrogen receptor-mediated signaling pathways in the endometrial. E2 promotes endometrial growth, while progesterone and other progesterone hormones block endometrial growth and promote differentiation. E2 mediates its biological response by binding to ERs *via* genomic and non-genomic pathways. There are 3 main mechanisms of genomic regulation. Firstly, in genomic regulation, E2 ligands passively enter the cells by diffusion. ERα and ERβ are located in the cytosol. The binding of E2 to the ER promotes the formation of dimers, enters the nucleus and is directly binding to EREs, or to transcription factors which regulate transcription of its target genes. Secondly, the nonclassical pathway involves binding the E2-bound ER to TFs that are already bound to the DNA. The third mechanism is hormone-independent. The ER can regulate E2 responses by activating the signaling of growth factors *via* the phosphorylation of different serine (118/167) residues on the receptor. In non-genomic regulation, binding of E2 to ERs and GPR30 at the plasma membrane leads to various nongenomic responses, such as calcium signaling, PKC, and cAMP/PKA pathways. E2, 17β-estradiol; ER, estrogen receptor; GPER, G-protein-coupled estrogen receptor; EGFR, epidermal growth factor receptor; PKA, protein kinase A; SF-1, steroidogenic factor 1; TNFα, tumor necrosis factor α; PR, progesterone receptor; MAPK, mitogen-activated protein kinases; PI3K, phosphoinositide-3-kinase. ERE, estrogen response element.

ERα and ERβ are members of the nuclear receptor (NR) superfamily and consist of five domains (A/B, DNA-binding; D, ligand-binding; and F) (**Figure 1**). The structurally distinct amino-terminal A/B domains share 17% amino-acid identity between the ERs, acting as ligand-independent activation function 1 (AF-1); AF-1 is involved in both inter-molecular and intra-molecular interactions as well as in activating gene transcription (2). The near-identical central C region (97% amino-acid identity) is the DNA-binding domain that allows ERs to dimerize. Acting as a flexible hinge, the D domain (36% amino-acid identity) contains a nuclear localization signal, is important for receptor dimerization and binding to chaperone heat-shock proteins and links the C domain to the multifunctional carboxyl-terminal (E) domain. The E domain, also known as the ligand-binding domain (LBD), shares 56% amino-acid identity between the ERs and is a globular region that comprises an 17β-estradiol (E2)-binding site, a dimerization interface (homo- and heterodimerization), and ligand-dependent coregulator interaction activity (activation function 2, AF-2); the LBD works synergistically with the amino-terminal domain to regulate gene transcription (3–6). The F domain shares 18% amino-acid identity between the ERs and is located at the extreme carboxyl-terminus of the receptors. In ERα, the F domain appears to modulate transcriptional activity, co-activator interactions, dimerization, and stability; however, its role in ERβ is unclear (7–9).

The uterus reacts to cyclical changes in estrogen and progesterone levels to prepare the embryo for implantation. Most known estrogen effects are mediated by ERα and ERβ, which regulate classical hormone signaling pathways (10). Both ERα and ERβ are expressed in the murine uterus (11). ER dimers bind to estrogen response elements (EREs) in the promoter regions of target genes, initiating the recruitment of co-activators, co-suppressors, and chromatin remodeling factors to activate or inhibit transcription of target genes. Despite the presence of similarities in initiation and termination, the ERE-dependent signaling pathway substantially differs between ERα and ERβ in terms of the mode and extent of transcription, presumably because their distinct amino-termini influence the magnitude of transcriptional responses (6, 12, 13). The amino-terminal A/B domains of ERα include the ligand-independent AF-1, while the LBD includes the ligand-dependent AF-2. Both AF-1 and AF-2 of ERα are necessary for uterine physiology because their functional integration is required to mediate transcription at full capacity in response to E2 in a tissue-specific manner (14, 15). In contrast, ERβ displays a lower affinity for ERE binding and considerably lower transcriptional activity in the E2-induced ERE-dependent genomic signaling pathway; moreover, it interacts with a different set of proteins, presumably because of the absence of AF-1 in its amino-terminus (6, 16). The N-terminal domain of ERβ is much shorter than the corresponding domain of ERα. Moreover, the amino-terminus of ERβ has been proven to impair receptor-ERE interactions and does not interact with the carboxyl-terminus (17, 18).

In the nonclassical pathway of estrogen action, ER can regulate genes that lack a canonical ERE. ER can activate or suppress gene expression by interacting with other transcription factors, such as activator protein-1 (AP-1), specificity protein-1 (SP-1), nuclear factor kappa-B (NF-κB) (19, 20). Jun and Fos are members of the AP-1 transcription factor family that can form Jun-Jun or Jun-Fos dimers; their basic regions can interact with a consensus sequence known as the TPA response element (21). Selective estrogen receptor modulators (SERMs) and selective estrogen receptor downregulators (SERDs) can activate the transcriptional responses mediated by ERα at TPA response element sites, while they cannot activate transcriptional responses mediated by ERβ (22–24). Another transcription factor, SP-1, binds to a consensus sequence known as the GC box element. ERα has been shown to tether to GC box response element-bound SP-1, for which the amino-terminus of ERα is critical (25). In addition, in ligand-independent genomic events, ER activates growth factor signaling by phosphorylating different serine (118/167) residues on the receptor, thereby regulating the E2 response.

Beginning in the 1990s, there was increasing evidence that rapid modulation (within seconds or minutes) of estrogen-mediated signaling pathways occurs *via* a subpopulation of ERs located in or adjacent to the plasma membrane; these non-genomic effects induced by E2 were determined by the observation that exposure of uterine or ovarian cells to E2 could rapidly induce ion influx, cyclic adenosine monophosphate (cAMP) production, and phosphoinositide-3-kinase (PI3K) activation (26–30). In the 2000s, membrane ERs were characterized; these primarily consisted of GPR30/GPER, ER-X, and the Gq membrane ER (31–33).

Among all membrane ERs, GPER has been recognized as the major mediator of the rapid cellular effects of E2 because stimulating GPER activates metalloproteinases and induces the release of heparin-binding epidermal growth factor, which binds and activates epidermal growth factor receptor (EGFR); this interaction leads to the activation of downstream signaling molecules such as extracellular signal-regulated kinase 1/2 (ERK1/2), followed by the production of cyclic cAMP, intracellular calcium mobilization, and PI3K activation (34–37). Additionally, GPER indirectly regulates transcriptional activity through signaling mechanisms that involve cAMP, ERK, and PI3K (38) (**Figure 1**).

Regarding targeted therapies for GPER, the first GPER-selective agonist G-1 was identified in 2006. Subsequently, a GPER-selective antagonist G-15 was identified in 2009, followed in 2011 by G-36, a more selective GPER antagonist than G-15 (39, 40). Fulvestrant is a SERD that causes degradation or downregulation of ER, but acts as a GPER agonist (41).

## Transcriptional Control by ERs

E2 activity is mediated by the two classical nuclear hormone receptors, ERα and ERβ. Transcriptional regulation arises from the direct interaction of the ER with components of cellular transcriptional machinery. Cells can express a set of coregulators that can enhance or decrease the transcriptional activity of steroid hormone receptors, called ER coactivators and corepressors, respectively. Coregulators are crucial for ER transcriptional activity and have led us to recognize that there is a great deal of sophistication in transcriptional regulation. ER is able to bind to cofactors through the AF-1 and AF-2 domains, acting synergistically in the recruitment of coregulators. Coregulators are both targets and propagators of posttranslational modification codes and are involved in many steps of the gene expression process, including chromatin modification and remodeling, transcription initiation, elongation of RNA chains, mRNA splicing, mRNA translation, miRNA processing, and degradation of the activated NR coregulator complexes (42).

Approximately 200 coactivators play a central role in NR-mediated promotion of gene expression and are associated with cancer and other diseases. Many additional ERα coregulators have been found, although little is known about Erβ (43). Investigation of coregulators of ERs began in the 1990s and the first identified ERα coregulator was called steroid receptor co-activator (SRC-1). Co-expression of SRC-1 reverses the ability of ER to inhibit human progesterone receptor (PR) activation (44). Coregulators of ERα include the SRC/p160 group, histone acetyltransferase cAMP response element binding protein (CREB)/p300, ATP-dependent chromatin remodeling complexes such as SWI/SNF, E3 ubiquitin protein ligases, and steroid RNA activator (45). Then transcriptional corepressors have the NR corepressor and thyroid hormone receptors (46, 47). Therefore, nuclear ERs play a variety of roles and functions in different cells and tissues that are mediated by various intermediaries and differential utilization of coregulators (48).

From studies of cancer cells, we have learned that a large number of coregulators have specific structural motifs that affect their contact with the ER ligand binding domain. The ER crystal structure suggests that helix 12 lays across the ligand binding pocket when binding agonists, resulting in a surface suitable for the binding of LXXLL motifs found on coregulators. Conversely, we know that co-inhibitory factors block ER-mediated gene transcription by directly interacting with unbound ERs, using their corepressor NR box or by competing with coactivators (49). It has also been reported that some post-translational modifications, such as phosphorylation, methylation, ubiquitination, SUMOylation and acetylation, can affect the action of coregulatory factors that target gene expression (50, 51).

## The Role of ERs in Endometrial Physiology

The dynamic expression pattern of steroid hormones and their receptors in the endometrium during the menstrual cycle has been established and drive cyclical growth, shedding, and regeneration of the endometrium. During the follicular phase of the menstrual cycle, estrogen, working through ERs, induces growth of the endometrium, producing a measurable thickening of the mucosa (52). After ovulation, the corpus luteum continues to produce a large amount of estrogen ligand in addition to progesterone. The presence of progesterone inhibits estrogen-induced endometrial growth and transforms the endometrium into a receptive state for blastocyst implantation (1). Physiologically, progesterone plays an important role in preventing endometrial hyperplasia (EH) (53). In animal models, high levels of estrogen without progesterone interference can lead to EH or cancer, suggesting that the balance between estrogens and progestogens is often dominated by estrogens during cancer formation (54, 55) (**Figure 1**).

During postnatal development, the uterus undergoes a unique pattern of changes in ER expression. ERα is weak and the proliferative activity is high from postnatal days 1 to 7 in the uterine stroma. In contrast, the cell proliferation rate is decreased from day 7 to puberty, but ERα expression in the epithelial and stroma cells is increased (56). The low rate of proliferation in the prepubertal period is due to estrogen levels being too low to activate ERα (57, 58). However, in the proliferative phase, ERα immunoreactivity is significantly stronger than ERβ immunoreactivity in the nuclei of epithelial, stromal, and muscle cells, suggesting ERα mRNA expression is dominant in the human uterus. Immunohistochemical localization revealed increased expression of ERα and ERβ in the late proliferative and early secretory phases, and then a decrease in the mid-late phase (59). ERβ is the main ER subtype expressed in the endometrial stroma in the late secretory phase (60). ERα and ERβ are differentially expressed in endometrial vascular endothelial cells and perivascular cells surrounding endometrial blood vessels. Monoclonal antibodies and immunocytochemistry have shown that ERα is localized to muscle cells of uterine arteries (61). Analysis of immunostaining confirmed that endometrial endothelial cells only express ERβ, which may be the target of selective agonists or antagonists for the ERβ subtype (59) (**Table 1**). It is notable that nonradioactive *in situ* hybridization confirmed that ERα and ERβ were localized in both epithelial and stromal cells of endometriotic tissues (66) (**Figure 1**).

**Table 1:** ER expression by uterine phase.

Tissue	Specific location	Period	Method	Results	References
ERα	The proliferative phase	The stroma and epithelial cells lining the glands	Real-time PCR; Immunocytochemistry	ERα mRNA was higher in the proliferative than in the secretory and menstrual phases	(59, 62, 63)
	The secretory phase	In the glands and stroma of the functionalis	Immunocytochemistry	ERα expression declined	(59)
	Days 7-27 of the normal cycle	Localized to perivascular smooth muscle Cells in nonpregnant endometrium	Monoclonal antibodies; immunocytochemistry	ERα was observed in muscle cells of uterine arteries	(59, 61)
ERβ	The proliferative phase	Predominantly in glandular epithelial cells	Using non-radioactive in-situ hybridization	ERβ mRNA concentrations were lower than ERα mRNA.	(64)
	The secretory phase	In nuclei of the glands and stroma	Real-time PCR; Immunocytochemistry	The mRNA expression of ERβ had a peak in the late secretory phase; ERβ declined much more in the glands than in the stroma	(59, 62)
	The proliferative and secretory phases of the menstrual cycle	In the endothelial cells	Immunohistochemistry	only ERβ was present in the endothelial cell population	(59)
GPER	The proliferative phase	In epithelium cells and stromal cells (epithelial cells are the main source of GPER mRNA)	Real-time PCR; Western blot; *In situ* hybridization;	High content (GPER predominantly localized in the mid- and late-proliferative phase)	(62)
	The secretory phase	In the stroma	Real-time PCR;	Low content (GPER dropped rapidly to low levels in the early secretory phase)	(62)
	The menstrual phase	In the stroma	Real-time PCR;	Low content	(62)
	In early pregnancy decidua phase	In glandular and luminal epithelium, and in the stroma	Real-time PCR; Western blot; Immunohistochemistry	Low content	(62)
	Myometrium	localized in the plasma membrane and in some areas colocalized with caveolae in myometrial smooth muscle cells	RT-PCR; Western blotting; Immunocytochemistry	concentrations of GPER mRNA and protein did not change across the not-in-labor to in-labor continuum	(65)

ER, estrogen receptor; GPER, G-protein-coupled estrogen receptor; RT-PCR, reverse transcription polymerase chain reaction.

The plasticity of the human endometrium is beyond other tissues, and it can still respond to steroid hormones and induce endometrial cycles after menopause. The post-menopausal endometrium has low circulating estrogen level and lacks progesterone, while androgen levels from adrenal glands are relatively unchanged. ERβ mRNA expression in the myometrium of postmenopausal women is significantly higher than in premenopausal women, while ERα expression is decreased (67). Thirty-one postmenopausal women underwent combined treatment with E2 and testosterone, which increased AR and ERβ expression in the endometrium (68). In contrast, levels of ERα and PRs in the endometrium were upregulated after estrogen-alone treatment (68). This study indicated that the antiproliferative effect of androgen treatment in the endometrium is associated with increased ERβ expression and that ERα may promote endometrial proliferation.

In humans, several studies have reported GPER mRNA and protein expression in the uterine epithelia (epithelial and stromal cells), endometrium, myometrium, and early pregnancy decidua (62, 65, 69). Real-time PCR and immunohistochemistry results showed that GPER mRNA was highly expressed in glandular epithelial cells in the mid- and late- proliferative phase, higher than during the secretory and menstrual phases. GPER expression levels were also found to decrease rapidly from the early secretory phase and remained low until the end of the cycle (62). In decidual tissue, GPER mRNA expression was localized in both the glandular and luminal epithelium and in the stroma (62). GPER activation enhanced contractile responses to oxytocin in the myometrium. These findings suggest that GPER may be involved in the physiological changes in human uterine activity during pregnancy (65). In general, several studies using cell and animal models have highlighted the importance of ER signaling in female reproduction. GPER is involved in cyclic alterations of endometrial estrogen action, and high GPER transcript levels were observed in the eutopic endometrium during the proliferative phase, whereas higher GPER mRNA expression has been shown during the secretory phase (70) (**Table 1**). GPER is also found in the endometrium of women with endometriosis (71).

## Roles of ERs in the Endometrium

It is currently thought that E2-induced uterine epithelial cell proliferation is mediated by stromal ERα. ERα knockout mice showed no expression of estrogen responsive genes in the uterus, and the basic levels of PR mRNA in the αERKO uterus were equal to those in wild type mice (72). The levels of PR protein in the uterus of ERα knockout mice were at 60% of the level measured in a wild-type uterus, which is enough to induce the genomic responses mediated by progesterone but not enough to support embryo implantation (73). These observations suggest that ERα modulation of PR levels is not necessary for progesterone action in ERα knockout mouse uterus (**Table 2**).

**Table 2:** Summary of mouse models involving ERα and ERβ.

Organisms	Year	Type	Fertility	levels of E2	Notes	Reference
Mouse model	1995	ERαKO	NA	serum levels of E2 in the ERαKO female are more than 10-fold higher than those in the wild type	increased DNA synthesis, and transcription of the PR, lactoferrin, and glucose-6-phosphate dehydrogenase genes	(72)
	2000	ERβ^−/−^ mice	poor reproductive capacity	NA	enlargement of the lumen; increase in volume and protein content of uterine secretion; induction of the luminal epithelial secretory protein	(74)
	2000	ERβ^−/−^ mice	exhibit variable degrees of subfertility.	NA	reproductive tract normal	(75)
	2002; 2006	NERKI mice	the heterozygous NERKI females (AA/+) are described to be infertile;	steroid hormone levels are similar to wild-type females	have grossly enlarged uteri with cystic hyperplasia	(76, 77)
	2003	immature female mice were treated with ER subtype-selective agonist	NA	NA	inhibited PR and AR mRNA and protein expression	(78)
	2006	ERβ^−/−^ mice	NA	NA	hyperproliferation and loss of differentiation in the uterine epithelium	(79)
	2009; 2014	EAAE mouse	infertility; females heterozygous for the EAAE ERα mutations are fertile	NA	the inability of E2 to induce uterine epithelial proliferation; has an ERα null–like phenotype; with impaired uterine growth and transcriptional activity; the hypoplastic uteri	(80, 81)
	2010	UtEpiαERKO	infertile	NA	the uterine epithelial E2-specific loss of response; increased uterine apoptosis	(82)
	2011	AF-2-mutated ERα knock-in (AF2ERKI)	infertile	high serum E2 levels	have hypoplastic uterine tissue and rudimentary mammary glands similar to ERαKO mice	(15)
	2013	mice lacking ERαAF-1 (ERα*AF-1^0^*)	NA	NA	ERαAF-1 is required for E2-induced uterine epithelial cell proliferation	(83)

NA, not available or not assessed; ER, estrogen receptor; E2, estradiol; PR, progesterone receptor; AR, androgen receptor.

ERs are dependent on AF-1 in the N-terminal domain and AF-2 in the C-terminal LBD to induce the specific conformational changes that are required for ER transcriptional activity (**Figure 1**). In the model of AF-2-mutated ERα knock-in (AF2ERKI), AF2ERKI homozygote female mice have hypoplastic uterine tissue and rudimentary mammary glands that are indistinguishable from ERα knockout mice (15). ERα AF-2 has been demonstrated to play a crucial role in the endometrial proliferative effect of E2. Furthermore, Abot et al. investigated the role of ERα AF-1 in the regulation of gene transcription and cell proliferation in the uterus. Targeted deletion of AF-1 in mice showed normal uterine development but delayed response to E2 (83). Nevertheless, in a study using the “EAAE” mouse, which has more severe DNA-binding domain mutations in ERα, uterine epithelial proliferation could not be induced through the estrogen signaling pathway, manifesting an ERα null-like phenotype with impaired uterine growth and transcriptional activity (80, 81) (**Table 2**). The ERα (EAAE/EAAE) mouse suggested that the DBD is necessary for estrogen action and the LBD is insufficient.

Nonclassical ERα knock-in (NERKI) mice with an ERα mutated at the DNA recognition helix that disrupts DNA binding but leaves nonclassical signaling intact have also been developed (**Table 2**). The uteri of NERKI mice are larger than those of ERα knockout mice but smaller than those of wild type mice. NERKI mice also have defective ovulation and underdeveloped mammary glands (76). The NERKI mice indicate that nonclassical ERα signaling plays a critical role in uterine growth and development, which is beneficial to restore the proliferation of luminal epithelial cells (77).

By establishing a uterine epithelial-specific ERα knockout (UtEpiαERKO) mouse line, it was found that while female UtEpiαERKO mice were infertile, they had regular estrous cycles and complete follicular development stages, indicating ovulation (82) (**Table 2**). Embryonic implantation was not observed in the uterus after natural mating or embryo transfer, suggesting that ERα in the uterine epithelium is necessary for embryo receptivity. E2 treatment to UtEpiαERKO mice stimulated epithelial cell growth, but apoptosis in the epithelial cells significantly increased compared with wild type mice. These studies might help to determine how the proliferation of uterine epithelial is mediated by ERβ in the stroma, while uterine epithelial-derived ERα is required subsequent to proliferation to prevent epithelial apoptosis ensuring the full uterine epithelial response (82).

Despite ERα being the predominant ER in the adult rodent uterus, transcripts encoding ERβ have also been detected in wild type and ERα^−/−^ murine uteri (11). The uteri of untreated ERβ^−/−^ mice exhibit exaggerated responsiveness to E2, as indicated by enlargement of the lumen, increased volume and protein content of uterine secretions, and induction of the luminal epithelial secretory protein (74). The increased cell proliferation and exaggerated response to E2 in ERβ^-/-^ mice suggest ERβ inhibits ERα function, resulting in an anti-proliferative function in the immature uterus. ERβ can act as a regulator of ERα-mediated gene transcription in the uterus, alternatively, it is responsible for downregulating PR in the luminal epithelium. Paradoxically, there is evidence that ERβ can partially compensate for loss of ERα in the reproductive tract, as the uterine phenotype of ERαβ double knockout mice is similar to the aggravated uterine phenotype of ERα knockout mice, whereas the reproductive tract of ERβ knockout mice appears normal (75) (**Table 2**). Meanwhile, the uterine epithelium of ERβ^−/−^ mice showed hyperproliferation and loss of differentiation (79). This suggests that the absence of ERβ predisposes the uterus to abnormal endometrial proliferation. Fully mapping ERβ expression in the endometrium may be useful in identifying women at higher risk of EH.

A study examining the potential synergistic regulation of gene expression and uterine growth by the two receptors for estradiol, ERα and ERβ, using ER subtype-selective agonist ligands showed that the ERβ agonist diarylpropionitrile did not increase uterine weight or luminal epithelial cell proliferation, but inhibited PR and AR mRNA and protein expression (78) (**Table 2**). Additionally, ERβ can modulate ERα activity in a response-specific and dose-dependent manner (78).

The structure of GPER is dramatically different from that of ERα and ERβ. No obvious developmental or functional defects have been observed in the reproductive organs of GPER knockout mice (84–86). Treating wild-type mice with G-1 stimulates uterine epithelial proliferation despite a lower potency relative to that of E2; conversely, blocking GPER with G-15 reduces the E2-mediated proliferative response by approximately 50% (40). Stable knockdown of GPER substantially eliminated the tumor growth induced by autocrine motility factor (AMF) in EC, with significantly longer survival times in tumor-bearing mice (87). Conversely, Gao et al. demonstrated that in an ovariectomized mouse uterus, GPER activation by high-concentration G-1 altered the expression of E2-dependent uterine genes and mediated inhibition of ERK1/2 and phosphorylation of ERα (Ser118) in the stromal compartment; thus, they concluded that GPER is a negative regulator of ERα-dependent uterine growth in response to E2, suggesting an interaction between non-genomic and genomic ERs (69).

Estrogen plays a different role in embryo implantation and angiogenesis. This suggests that although studies in mouse models provide evidence for the role of ovarian steroid hormones in regulating uterine function, extrapolation of mouse endometrial estrogen findings to human conditions needs to be considered carefully. Despite their compelling results, many of the reviewed studies were limited by a lack of replication, small sample sizes, retrospective designs, publication bias, and/or the use of non-standardized tools to diagnose conditions.

## The Endometrium and ERs

### Endometriosis

Endometriosis is the presence of endometrial cells outside the uterine cavity, which can invade local tissues and cause severe inflammation and adhesions. Approximately 15% of infertile women are reported to have endometriosis, although the true prevalence of endometriosis is unclear (88). Multiple cellular and molecular signaling pathways are likely to be involved in the pathogenesis of endometriosis.

**Figure 2:**
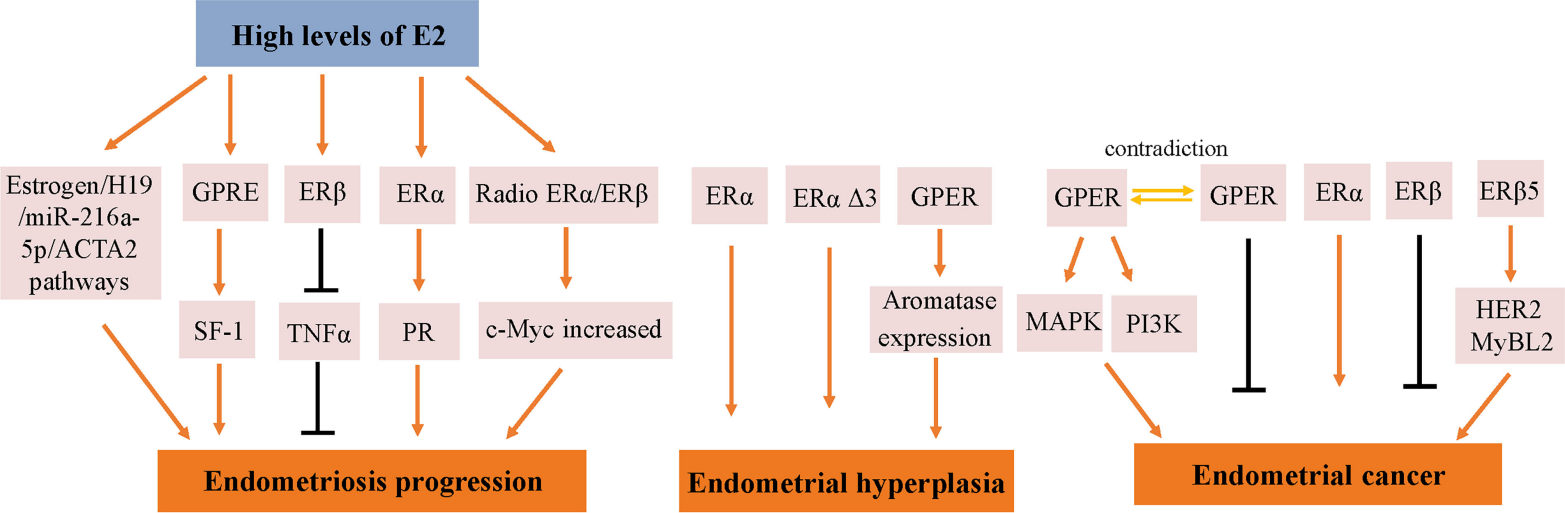
Molecular pathways regulated by ER in endometrial diseases. E2, estradiol; ER, estrogen receptor; GPER, G-protein-coupled estrogen receptor; SF-1, steroidogenic factor 1; TNFα, tumor necrosis factor α; PR, progesterone receptor; MAPK, mitogen-activated protein kinases; PI3K, phosphoinositide-3-kinase.

Endometrial development and function are highly dependent on the cyclic secretion of sex steroid hormones and the expression of their cognate receptors (89). The endometrial cell types that are primarily targeted by steroid hormones include epithelial and stromal cells. Proliferation and differentiation of the endometrium are regulated by estrogens (90). Endometriosis typically involves higher levels of E2 than observed in a normal endometrium, which is due to higher gene expression levels of aromatase and 17β-hydroxysteroid dehydrogenase type 1 (91); the higher levels of E2 result in increased E2 binding and activation of ERs in endometriotic tissues, thereby stimulating estrogen-dependent growth. These higher levels of local E2 activity could contribute to the proliferation of endometriotic tissues (92). Estrogen-mediated changes in cell signaling presumably have important implications for the pathogenesis of endometriosis (93). For example, the invasion and migration of eutopic endometrial endometriosis stromal cells is regulated by the estrogen/H19/miR-216a-5p/ACTA2 axis (94). Alternatively, studies have implicated that tamoxifen and the phytoestrogen genistein can induce steroidogenic factor 1 (SF-1) target gene aromatase expression in a GPER-dependent manner to promote the proliferation of Ishikawa cells. Based on this finding, we hypothesized that GPRE/SF-1 may promote endometriosis by increasing local estrogen concentrations and mediating the proliferation of synthetic estrogens in combination with classical ER signaling (95) (**Figure 2**).

Estrogen activity is mediated *via* genomic pathways including nuclear ERα/β, as well as by more rapid, non-genomic pathways, such as ERα36 and GPER (96). Elevated estrogen promotes the expression of ERα and ERβ, which reach their highest levels in the late proliferation phase (97); however, aberrant levels of ERs are observed in women with endometriosis. Compared with endometrial tissue, ERβ mRNA and protein levels were more than 100-fold increased, while the levels of ERα were several times lower (98); inhibiting the enhanced ERβ activity *via* an ERβ-selective antagonist also suppressed the growth of ectopic lesions in mice (99). Notably, ERβ activity stimulates endometriotic progression: ERβ inhibits tumor necrosis factor α (TNFα)-induced apoptosis through interactions with apoptotic mechanisms to avoid endogenous immune surveillance of surviving cells (99). ERβ directly induces Ras-like estrogen-regulated growth inhibitor gene expression in an estrogen-dependent manner to enhance the proliferative activity of endometriotic tissues (100). The role of ERβ is presumably more complicated, because greatly elevated levels of ERβ are present in both the nucleus and cytoplasm of endometriotic tissues (101).

It remains controversial whether ERα exhibits an endometriotic tissue-specific pattern (102). Studies of ERα knockout mice with endometriosis have shown that ERα causes cell adhesion and proliferation and that it regulates inflammatory signaling in ectopic lesions (103). E2 increases the expression of PRs mainly through ERα activation, thereby mediating the effects of progesterone on the endometrium and triggering the secretory phase of endometrial circulation. Estrogen responsiveness is considerably complex, as indicated by the results obtained from experiments in mice and the findings in human tissues contain splice-form variants of ERα and ERβ. Moreover, the differential effects of estrogen on endometrial cells may depend on the total amount of cellular ERs and/or the ratio of ERα to ERβ (104).

### EH

EH is a uterine pathology that involves a continuum of morphologic alterations that range from mild, reversible glandular hyperplasia to direct cancer precursors. Compared with the normal proliferative endometrium, the predominant characterization of EH is an increased endometrial gland-to-stroma ratio. During the reproductive period, the risk of EH is increased by conditions associated with intermittent or anovulation, such as Polycystic ovary syndrome. After menopause, when ovulation stops, EH is more common in women with increased circulating estrogen levels.

During the normal reproductive cycle, ERα expression in uterine epithelial cells is downregulated in the secretory phase. Hu et al. assessed the expression of ERα and ERβ in 114 patients using immunohistochemistry (105). The results indicated that from normal proliferation to simple and complex hyperplasia, the expression of ERα increased, while the expression of ERβ showed no significant change. Other studies have also revealed similar results. As early as 2003, Uchikawa et al. detected the expression levels of ERs in 20 normal endometrium samples, 36 hyperplastic endometrium samples, and 58 malignant endometrium samples, and found that ERα expression was increased in EHs compared with normal endometrium samples (106). In 2005, Bircan et al. described that ERα levels were significantly higher in EH than in the normal secretory endometrium (107).

However, different variants have different protein activities and different regulatory functions on ER signaling. ERα Δ3 lacks part of the DNA binding domain of exon 3 and inhibits estrogen-dependent transcription activation in a dominant negative way, which may potentially affect ERα signal transduction (108). The Δ3 variation is detected in prolactinoma, EH, and breast cancer, but not in the normal pituitary, normal endometrium, or endometrial cancer (EC) (109–111). For example, this variant was found in 19 of 21 EH cases, but not in any of 29 EC cases (110). Additionally, ERα Δ3 expression is reduced more than 30-fold in breast cancer compared with in the normal breast epithelium (112). It is speculated that the dominant negative activity of Δ3 may decrease normal estrogen signaling, and thus interfere with tumor progression and growth (**Figure 2**).

GPER is highly expressed in abnormal EH, and its expression trend follows that of ERα, which is gradually increased from the normal proliferative endometrium to simple EH, and then to a maximum in complex EH (**Figure 2**). This could imply that in normal and benign proliferation, GPER expression increases proportionally due to the induction of GPER by ERα (71, 113). Aromatase activity is not detected in the normal endometrium but is highly expressed in endometriosis and malignant endometrium cells (114, 115). Surprisingly, GPER activation increased aromatase expression in both endometriosis and malignant endometriosis cells (95). Thus, we speculate that the mechanism by which GPER regulates the growth of abnormal endometrial cells might be that it induces the expression of aromatase, increasing the synthesis of intracellular estrogen. In turn, estrogen activates intracellular GPER by the intracellular pathway, further increasing the abnormal proliferation rate of cells.

### EC

EC is an estrogen-dependent malignancy. The administration of estrogen alone for an extended period can increase the risk of EC in postmenopausal women. Some endometrial atypical hyperplasias can evolve into EC over a long period of time. EC is subdivided into two types on the basis of histopathology. Type I endometrial tumors, also known as low-grade endometrioid, which make up account for 75% of endometrial cases, are usually associated with high levels of ERα (116). Type II tumors include high-grade endometrioid tumors, serous tumors, clear cell tumors, carcinosarcomas, and mixed histology tumors. Carcinomas can typically be classified into two types according to their estrogenic state, type 1 (ER-positive EC) and type 2 (ER-negative EC).

In a previous study, the expression of GPER, estrogen, progesterone, EGFR, and Ki-67 in 47 EC patients treated between 1997 and 2001 based on early immunohistochemical methods showed that GPER was overexpressed in high-risk EC patients and was negatively correlated with PR expression (117). E2 and G-1 trigger the mitogen-activated protein kinase (MAPK) pathway in ER-negative KLE cells and ER-positive RL95-2 cells, which require GPER involvement (118). Li et al. investigated the relationship between AMF and GPER in EC (87). This mechanistic study demonstrated that the interaction between AMF and GPER activates PI3K signaling, which in turn accelerates the growth of EC cells (**Figure 2**). However, there have been inconsistent results regarding the expression of GPER in EC. In 2012, Krakstad et al. found decreased GPER mRNA and protein levels and increased ERα levels in high grade endometrial carcinoma, supporting the association between GPER loss and disease progression from primary to metastatic lesions (119). Furthermore, in another study, GPER expression in EC cells was found to be lower than in normal endometrial samples. Treating GPER-positive EC cells with the GPER agonist G-1 resulted in significant growth reduction, suggesting that GPER mRNA might be sufficient to mediate the antiproliferative effects of its ligand in EC (120) (**Figure 2**). Due to its high expression and mitogenic role in other tissues and cancers, among the three ERs encoded in the human genome, ERα and ERβ are considered to be the major mediators of pro-growth estrogen signaling in EC cells.

The results of studies investigating the expression and role of ERβ in EC are not yet fully known. ERβ may play a suppressive role in EC. Immunohistochemical results have shown decreased ERβ mRNA and protein levels in endometrioid EC compared with in normal proliferative endometrium or adjacent normal endometrium from post-menopausal control women (121, 122). In addition, Zhang et al. indicated that in ERβ knockout mice, there is an unusual proliferation of cells in the uterus (74). Nevertheless, ERβ also shows a potential tumorigenic effect. A C-terminally altered ERβ isoform, ERβ5, is upregulated in endometrial carcinoma and is associated with the expression of oncogenes such as HER2 and MyBL2 (123, 124) (**Figure 2**).

Analysis of The Cancer Genome Atlas data showed that the mean expression level of ERα in EC was 2.9-fold higher than that of ERβ (125). The reason for this may be that EC mainly affects postmenopausal women, and higher gonadotropin levels in postmenopausal women may downregulate ERβ (126). Because ERβ acts as a dominant negative regulator of ERα, the postmenopausal endometrium may promote uterine cell proliferation through unopposed ERα action (74). In terms of analyzing the relationship between ERα/ERβ and the clinical characteristics of EC patients, it was found that ERα expression is higher in the early stages of EC and decreased in advanced EC (127). Thus, ERα may promote the progression of EC by interacting with estrogen in endometrial atypical hyperplasia and the early stages of EC.

It is well known that obesity is one of the most common risk factors for EC because androgens are converted to estrogen in adipose tissue. In fact, ER mutations are present in 5.8% of primary ECs (128). In EC, ERα mutations are associated with worse outcomes and less obesity, so mutations in ERα might explain why women with normal body and without other risk factors also develop EC (129). Evaluation of an EC cell model that includes that D538G mutation revealed that mutant ER has estrogen-independent activity as well as an expanded set of genomic binding sites (130). Mutation confers estrogen-independent activity to ER, which causes gene expression changes. Understanding the molecular and pathological effects of ER mutations in EC will further our knowledge of ER mutant disease and may provide treatment options for patients with ER mutant tumors.

ER is used as a regulatory cofactor to regulate gene expression, and different transcription factors may be responsible for controlling the genomic interactions of ER in EC cells. Motif analysis of endometrial cancer-specific ER-bound sites, along with gene expression analysis, revealed that ETV4, a member of the ETS family, overlaps with 45% of ER binding sites in Ishikawa cells (131). In a recent study reported using CRISPR/Cas9 to knockout ETV4 in EC cells, genetic deletion of ETV4 resulted in a large reduction of ER binding signal at the majority of bound loci across the genome, leading to an expected decrease in the transcriptional response to E2 treatment and thus reduced cells growth (132). Qi et al. found that estrogen regulates the histone acetylation hMOF expression through activating the PI3K/Akt and Ras–Raf–MEK–ERK signaling pathway to promote cell cycle progression in EC cells (133). Unfortunately, although the role of ER in the development of EC has been demonstrated in numerous studies, there are still many gaps in our knowledge of ER in EC.

## Treatments Targeting ERs

Hormone replacement therapy (HRT) has been used in menopausal women to relieve hot flashes, vaginal dryness, fluctuating emotions, irregular menses, chills, and sleeping problems. Pure antiestrogens represent an endocrine-targeted therapy for which the mechanism of action involves competition with ER ligands and ER downregulation. However, treating menopause symptoms with estrogen must be accompanied by a progestin component to avoid the effects of excess estrogen on the endometrium. Progesterone has been shown to downregulate ERs and stimulate direct PR-mediated effects that oppose estrogenic actions. In a postmenopausal estrogen/progestin interventions trial, women assigned to estrogen alone were more likely to develop simple, complex, or atypical hyperplasia. Combining conjugated equine estrogens with cyclic or continuous progestin protected the endometrium from hyperplastic changes associated with estrogen-only therapy (134). A small but not significant reduction in the risk of EC was observed in a sequential combination regimen with estrogen and progesterone (135). In the early 2000s, the Women’s Health Initiative raised numerous concerns regarding the use of hormone replacement therapy, as combined estrogen-progestin treatment was associated with a statistically significant increase in invasive breast cancer and in cardiovascular events after approximately five years of follow-up (136). Therefore, hormone replacement therapy use is now recommended to be relatively short-term (i.e., 3–5 years in post-menopausal women) and be administered at low doses; moreover, its use is very narrow and should be limited to women without a history of breast cancer and women who are not at increased risk of cardiovascular or thromboembolic disease (137). Consequently, novel ER modulators are necessary to maintain endocrine homeostasis.

**Figure 3:**
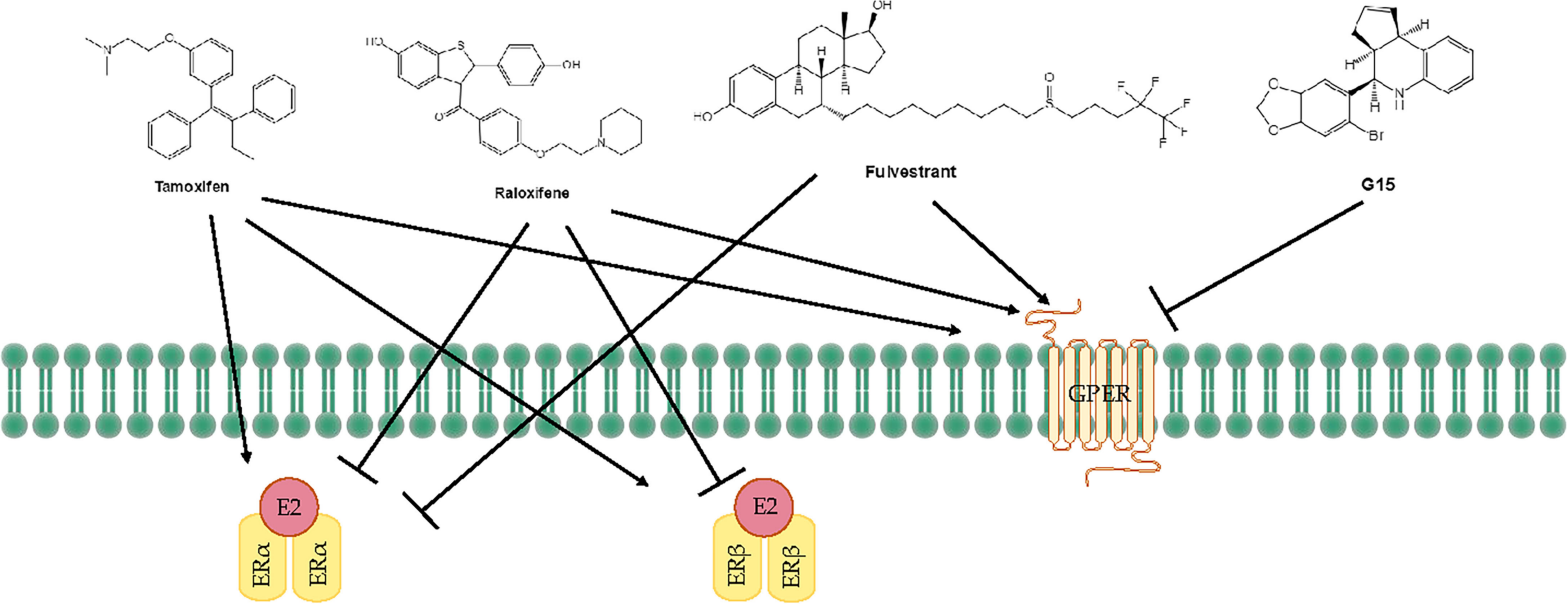
Treatments for endometrial diseases. ER, estrogen receptor; GPER, G-protein-coupled estrogen receptor; EGFR, epidermal growth factor receptor; ERE, estrogen response element; MMP, matrix metallopeptidase; LBD, ligand-binding domain; P, phosphorylation.

Tamoxifen acts as an agonist in most estrogen target tissues, presumably in association with differences in the expression of co-activator and co-repressor proteins in different tissues, which result in the formation of distinct complexes with ERs (6, 138) (**Figure 3**). Tamoxifen stimulates ER dependent gene regulation in the uterus (139). In endometrial cells, tamoxifen-bound ERα is able to recruit coactivator proteins and to initiate gene transcription. This differential recruitment of coactivators contributes to the tissue specificity of tamoxifen ERα function (140). However, the estrogen agonist effects on the endometrium from partial agonists cannot be neglected and can manifest as increased endometrial thickness, endometrial polyps, leiomyomas, and EC (141–143). Tamoxifen may promote cancer development by upregulating ERα, PR, vascular endothelial growth factor, EGFR, mechanical target of rapamycin, IGF-1R, and C-MYC in EC cells (144, 145). In a randomized trial, Fornander et al. found that among 931 postmenopausal patients treated with tamoxifen, there was a 6.4-fold increase in the incidence of EC compared with controls (146). This could be related to the dose of tamoxifen used (40 mg per day), which is higher than what was used by other trials. Similarly, Fisher et al. demonstrated a significant increase in EC severity among women treated with tamoxifen, with a relative risk of 7.5 (147). It has been suggested that tamoxifen treatment has a cancer-promoting effect *via* GPER, which significantly stimulates the proliferation of endometrial cells (148). Because tamoxifen has a selective antiestrogenic effect in breast cancer but an agonistic estrogenic effect in the bones and the uterus/EC, it is not suitable for use in the general population due to the increased incidence of EC.

The mechanism of action of raloxifene occurs through binding to ERα and ERβ. This binding results in activation and blockade of estrogenic pathways in tissues that express ERs. The crystal structures of the LBD of ER in complex with the endogenous estrogen, E2, and the selective antagonist raloxifene, have shown that they are all bind in the same site within the core of the domain, but raloxifene induces a transcriptionally inactive LBD conformation (149). Raloxifene has estrogenic antagonistic effects in the uterus and breast. ERα blockade using raloxifene indicated that E2 alters endometrial cell proliferation *via* ERα. The antagonistic effect of raloxifene on estradiol-treated endometrial epithelial Ishikawa cells has been demonstrated by the altered expression of genes such as HOXA 10, leukemia inhibitory factor, PR, and EMX2 (150). Notably, raloxifene does not exhibit the endometrial side effects observed with tamoxifen (151, 152). In estrogen-stimulated ovariectomized rat surgical models of endometriosis, treatment with 10 mg/kg raloxifene for 7 to 14 days resulted in reduced uterine volumes (153). Adult female rhesus monkeys with spontaneous endometriosis treated with 10 mg/kg/d raloxifene for 90 days showed degeneration of endometrial tissue and attenuated uterine volume (154). Although raloxifene does not induce breast tenderness, EH, or EC, it may augment the risk of thromboembolic disease (1/1000 cases per year) as well as hot flashes (in 4%–6% of cases). Furthermore, raloxifene reduces proliferative markers in the epithelium of lesions in rodent models but not in the stroma, while the stromal component is the major contributor to endometriotic lesions (155). Petrie et al. have demonstrated that GPER plays an important role in the estrogen-mediated signaling of the ERα^−^/ERβ^−^ EC cell line Hec50 and the SERM raloxifene is an agonist for GPER (156). In contrast to tamoxifen or raloxifene, antagonists such as fulvestrant (also known as SERDs) show complete antagonism. It completely inhibits estrogen-mediated changes in gene transcription and therefore has no agonist activity. The large side chain that originates from the B ring prevents H12 of ERα from docking in the agonist conformation, thereby preventing co-activator binding and transcriptional activation (149, 157) (**Figure 3**). Other effects of fulvestrant include inhibition of receptor dimerization and nucleocytoplasmic shuttling of the ER (158). Tamoxifen, raloxifene, or fulvestrant have been shown to be agonists of GPER. However, pharmacological inhibition of GPER activity *in vivo* prevents estrogen-mediated tumor growth, and the selective GPER antagonist G-15 retarded the growth of endometrial carcinoma (40) (**Figure 3**).

## Conclusion

This review provides a summary of the body of published systematic reviews and meta-analyses of the effects of different ERs on the endometrium. The endometrium is a dynamic tissue that undergoes proliferation, secretion, and menstrual during the menstrual cycle of the female reproductive age. The current findings show that unopposed endometrial exposure to estrogen increases the risk of EH and cancer and emphasize the importance of modulating ERs to control the development of endometrial diseases. Multiple variants of ER are involved in endometrial pathophysiology and signaling pathways. ERα promotes uterine cell proliferation and is strongly associated with an increased risk of EC, because it plays an important role in tumor development and metastasis by activating signaling pathways involved in promoting proliferation, resisting apoptosis, stimulating migration and invasion, and inducing angiogenesis. In contrast, the responses mediated by ERβ has a key opposite effect in the endometrium. GPER is normally expressed in the endometrial but is highly expressed in abnormal EH, whereas paradoxically expressed in patients with EC. More research is needed to elucidate the disease mechanisms that involve ERs.

It is well known that the identification of risk factors that are strongly associated with endometrial risk can help the identification of high-risk groups of women, which will benefit from targeted prevention strategies. The mechanisms of action of hormone therapy involves competition with the ER ligands and ER downregulation. Unfortunately, the role of HRT has been debated. Estrogen receptor modulators provide potential treatment for high-risk women. Among SERMs, tamoxifen therapy improves survival in Erα-positive primary and advanced breast cancer. However, in endometrial, many authors have confirmed that tamoxifen use may cause endometrial thickness and polyps (54, 159). Raloxifene, a second SERM, has a high affinity to ERα, with a relative binding affinity of 46% for human ERα compared with E2 (160). It has anti-estrogen effects on the uterus and protects the endometrium from hyperplasia and irregular bleeding caused by estrogen hyperstimulation. Fulvestrant is a non-agonistic ER antagonist that blocks the ER and inhibits the proliferative effects of estrogen on tumor cells. To better treat or cure endometrial disease, a deeper knowledge of the roles of ERα, ERβ or GPER and their interactions is required.

Although there are still many diseases for which estrogens have been implicated but the role of their receptors has not been elucidated. Endometrium-associated diseases may require simultaneous attacks on multiple targets or a systems approach for effective treatment. With the increased understanding of the molecular basis and the pathways related to specific disease progression, the era of molecularly targeted therapies has emerged as a most promising direction of research. To develop personalized therapies, the molecular regulation of endometriosis, EH and EC needs to be carefully studied. Because endometrial disease is essentially a hormone-dependent manifestation of high ER and PR expression, targeting ER may be a viable approach to develop novel treatment strategies for this disease. Screening of various compounds by molecular simulation can help to identify promising selective agonists or antagonists for the prevention and treatment of estrogen-affected endometrial diseases.

Further work is needed to develop new, more bioavailable SERMs/SERDs with better pharmacological properties, and therapies that inhibit all types of ERs. Furthermore, due to low bioavailability, it is expected to improve existing formulations to address the barriers to optimal SERM or SERD use and efficacy profiles.

## Author Contributions

KY, X-LX, and Z-YH reviewed the literature, wrote the manuscript, and designed the figures and tables. JL revised the draft. X-WF and S-LD made substantial contributions to the conception and design of the work and provided input into manuscript content and composition. All authors contributed to the article and approved the submitted version.

## Funding

This work was supported by National Nature Science Foundation Project of China (No. 32072722,31101714, and 31372307).

## Conflict of Interest

The authors declare that the research was conducted in the absence of any commercial or financial relationships that could be construed as a potential conflict of interest.

## Publisher’s Note

All claims expressed in this article are solely those of the authors and do not necessarily represent those of their affiliated organizations, or those of the publisher, the editors and the reviewers. Any product that may be evaluated in this article, or claim that may be made by its manufacturer, is not guaranteed or endorsed by the publisher.
